# The social media discourse of engaged partisans is toxic even when politics are irrelevant

**DOI:** 10.1093/pnasnexus/pgad325

**Published:** 2023-10-06

**Authors:** Michalis Mamakos, Eli J Finkel

**Affiliations:** Department of Psychology, Northwestern University, 2029 Sheridan Road, Evanston, IL 60208, USA; Department of Psychology, Northwestern University, 2029 Sheridan Road, Evanston, IL 60208, USA; Department of Management and Organizations, Kellogg School of Management, Northwestern University, 2211 Campus Drive, Evanston, IL 60208, USA

**Keywords:** political polarization, incivility, social media, echo chambers, computational social science

## Abstract

Prevailing theories of partisan incivility on social media suggest that it derives from disagreement about political issues or from status competition between groups. This study—which analyzes the commenting behavior of Reddit users across diverse cultural contexts (subreddits)—tests the alternative hypothesis that such incivility derives in large part from a selection effect: Toxic people are especially likely to opt into discourse in partisan contexts. First, we examined commenting behavior across over 9,000 unique cultural contexts (subreddits) and confirmed that discourse is indeed more toxic in partisan (e.g. *r/progressive*, *r/conservatives)* than in nonpartisan contexts (e.g. *r/movies*, *r/programming*). Next, we analyzed hundreds of millions of comments from over 6.3 million users and found robust evidence that: (i) the discourse of people whose behavior is especially toxic in partisan contexts is also especially toxic in nonpartisan contexts (i.e. people are not politics-only toxicity specialists); and (ii) when considering only nonpartisan contexts, the discourse of people who also comment in partisan contexts is more toxic than the discourse of people who do not. These effects were not driven by socialization processes whereby people overgeneralized toxic behavioral norms they had learned in partisan contexts. In contrast to speculation about the need for partisans to engage beyond their echo chambers, toxicity in nonpartisan contexts was higher among people who also comment in *both* left-wing and right-wing contexts (bilaterally engaged users) than among people who also comment in only left-wing *or* right-wing contexts (unilaterally engaged users). The discussion considers implications for democratic functioning and theories of polarization.

**Significance Statement**

Political discourse on social media is infamously uncivil. Prevailing explanations argue that such incivility is driven by differences in ideological or social-identity conflict—partisans are uncivil because the political stakes are so high. This report considers a different (albeit not contradictory) possibility—that online political discourse tends to be uncivil because the people who opt into such discourse are generally uncivil. Indeed, people who opt into political discourse tend to be especially toxic, *even when discussing nonpolitical topics in nonpartisan contexts*. Such individuals disproportionately dominate political discourse online, thereby undermining the public sphere as a venue for inclusive debate.

Partisan hatred is surging, both in the United States (1, 2) and in many other nations (3, 4). Such hatred, along with the associated anger, is linked to incivility toward opposing partisans (5), especially among those who are deeply engaged in politics (6–8). Indeed, animosity toward opposing partisans motivates political engagement on social media (9), where engaged partisans are especially likely to amplify moralized-emotional political content (10).

Why are such deeply engaged partisans so uncivil in their political discourse? Two theories prevail. The first focuses on political ideology, suggesting that the politically engaged are especially likely to perceive that opposing partisans hold unacceptable values and policy preferences (7, 11, 12). For example, comments on political blogs tend to be more uncivil insofar as the commenter holds more extreme views regarding the ideological social movement in question (e.g. Occupy Wall Street; (13)). The second focuses on social identity, suggesting that the politically engaged are especially likely to perceive their group as competing against opposing partisans for resources and status (14–17). For example, strong partisan identifiers hold particularly uncivil attitudes (18), and reactions on Facebook tend to be especially contemptuous (the “haha” reaction) insofar as the content of the post focuses on opposing partisans (9).

Both of these theories suggest that the political context is, for ideological or identarian reasons, a necessary condition for explaining the political incivility of engaged partisans. But neither of these theories offers predictions about incivility in contexts that are irrelevant to politics—contexts in which people gather to discuss, for example, movies, parenting, or computer programming.

The present report considers a different (albeit not contradictory) possibility, which we call the *troll hypothesis*: that online political discourse tends to be uncivil because the people who opt into such discourse are generally uncivil. Indeed, people who are more dispositionally disagreeable hold more negative views of opposing partisans (19); those who are more dispositionally aggressive engage in more aggressive political behavior and hold more violent partisan views (20). Recent articles have demonstrated consistency in hostile behavior in online and offline political discourse (21), and that when prompted to comment on posts related to politics, people who have online political activity are more likely to exhibit toxic behavior than people who do not (22). Although these prior studies have demonstrated self-selection effects related to the behavior of the politically involved in online environments, no research has investigated (i) within-person differences in hostility between partisan and nonpartisan contexts or (ii) between-person differences in hostility between engaged partisans (people with activity in partisan contexts) and the nonengaged (people without activity in partisan contexts) in contexts in which politics are irrelevant. Insofar as the incivility of engaged partisans results from broader dispositional tendencies toward aggressive behavior, such individuals are hypothesized to be uncivil in both partisan and nonpartisan contexts—and more uncivil than the nonengaged, *even when discussing nonpolitical topics in nonpartisan contexts*.

A compelling test of the troll hypothesis requires a study that affords two crucial comparisons. The first compares the behavior of engaged partisans in partisan vs. nonpartisan contexts to test whether people are *toxicity specialists* (i.e. only when politics are relevant) vs. *toxicity generalists* (i.e. in both political and nonpolitical contexts). The second compares the behavior of the engaged and the nonengaged in nonpartisan contexts to test whether engaged partisans are more toxic than the nonengaged when politics are irrelevant. Ideally, such a study would investigate not one or two of each type of context (partisan and nonpartisan), but thousands of them—and those contexts would be highly diverse in terms of their subject matter. Furthermore, the study should allow to test whether the behavior of engaged partisans is a product of dispositional tendencies vs. of socialization in partisan contexts.

Ideally, the study would also investigate such behavior in an important *public square*—a place where millions or billions of people come to introduce and debate societally important ideas. It would include both left-wing and right-wing cultural contexts to allow us to explore whether incivility in nonpartisan contexts varies as a function of whether engaged partisans comment on one side vs. both sides of the partisan divide (unilaterally vs. bilaterally engaged partisans). Scholars and social commentators have argued that a major cause of partisan toxicity is the emergence of an “echo chamber” phenomenon in which people encounter people and ideas that come disproportionately from their own side of the divide (e.g. Refs. (23–25)). However, a major study demonstrated that the political extremity of American partisans actually increased after people were assigned to see social-media posts from opposing partisans (26). In our study, participants were not randomly assigned to see posts from opposing partisans; rather they had the option of engaging in communities on one side vs. on both sides of the political divide. If the echo chambers hypothesis applies here, then the bilaterals should be less toxic than the unilaterals. In contrast, if the troll hypothesis applies here, then bilaterals should be more toxic, as dispositionally uncivil people are hypothesized to opt into political discourse—to jump into the fray—across the partisan divide.

To meet these criteria, we studied commenting behavior on Reddit from 2011 to 2022. Billions of people around the world use Reddit, which is also the fifth most-visited website in the United States, where it had 2.32 billion visits in March of 2023 alone.^1^ Compared to Facebook and Twitter, Reddit is much less dependent on algorithms that determine which information users are exposed to (27), which means that behavior on the platform is driven by user decisions to opt into a given context to make comments rather than being exposed to some contexts rather than others.

We began by considering which cultural contexts (subreddits) are political and which are nonpolitical. Politics can be relevant even in contexts that are not explicitly political, especially insofar as groups consisting of politically like-minded people adopt a worldview or style of discourse that leans left or right. Consequently, we employed both a *content* criterion and a *partisan segregation* criterion to establish a given context as *nonpartisan*: it must (i) focus on nonpolitical content and (ii) be populated about equally by people who tend to lean left vs. right. We operationalized the *partisan segregation* of each subreddit in terms of the extent to which the social networks of contributors to that subreddit overlapped with the contributors in left-wing vs. right-wing political subreddits (27). Some highly segregated subreddits are explicitly political (e.g. *r/hillaryclinton*, *r/The_Donald*), whereas others are ostensibly nonpolitical (e.g. *r/librarians*, *r/wrestling*)—but all of them are populated disproportionately with people who generally engage in either left-wing or right-wing social contexts. In this report, we define *engaged partisans* as users with activity in highly segregated subreddits, which may or may not be explicitly political content.

## Results

**Fig. 1.**
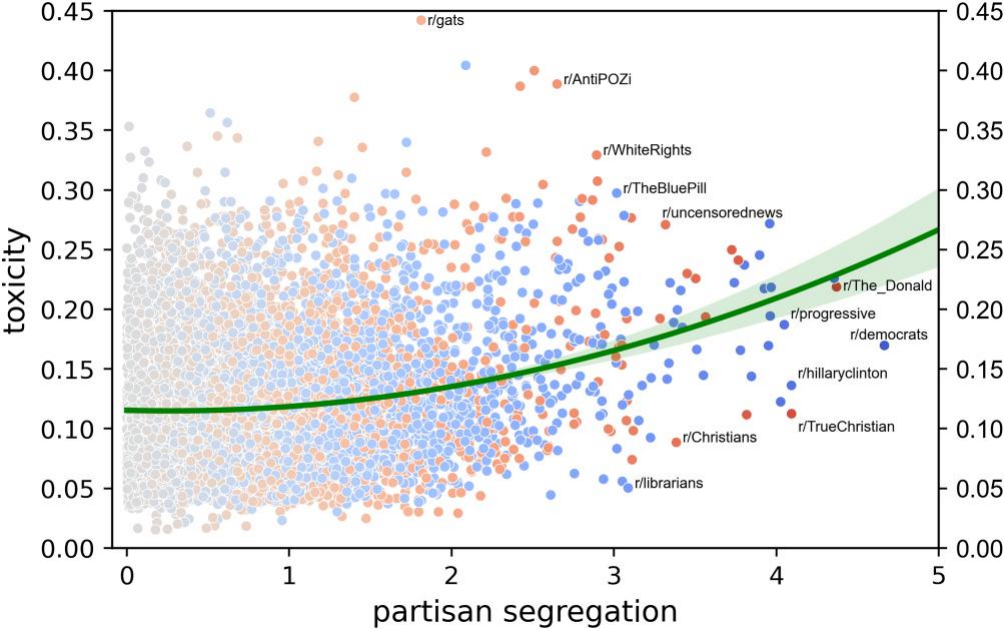
The toxicity and partisan segregation of 9,364 subreddits. The color of a dot (blue or red) indicates the partisan lean (left-wing or right-wing) of that subreddit.

In our first analysis, we assessed whether users’ commenting behavior is indeed more toxic in subreddits that are higher (vs. lower) in partisan segregation, operationalizing *toxicity* using Google's PerspectiveAPI classifier, which assesses the probability that a comment is “rude, disrespectful, or unreasonable and is likely to make someone leave a discussion” (28). Complementing research demonstrating that social-media discourse is more uncivil in contexts focusing on political than on nonpolitical content (29), we tested whether such discourse is more toxic in contexts disproportionately populated by partisans on one side of the political divide (regardless of the contexts’ content focus). A random sample of over 260 million comments from 9,364 subreddits (the substantially active of the 10,006 subreddits considered by Ref. (27)) revealed a quadratic effect of partisan segregation on toxicity (*β* = 0.21, *P* < 0.0001; the magnitude of this quadratic effect was virtually identical for both left-wing and right-wing subreddits, see Fig. S8 in *SI Appendix*). As hypothesized, the association of segregation with toxicity became increasingly positive at higher levels of segregation. As depicted in Fig. 1, partisan segregation and toxicity were largely unrelated in subreddits where segregation is modest, but these two variables were robustly linked in highly segregated subreddits. For example, for subreddits that are at least 2 *SD*s from the neutral point of 0, *r* = 0.25, *P* < 0.0001.

**Fig. 2.**
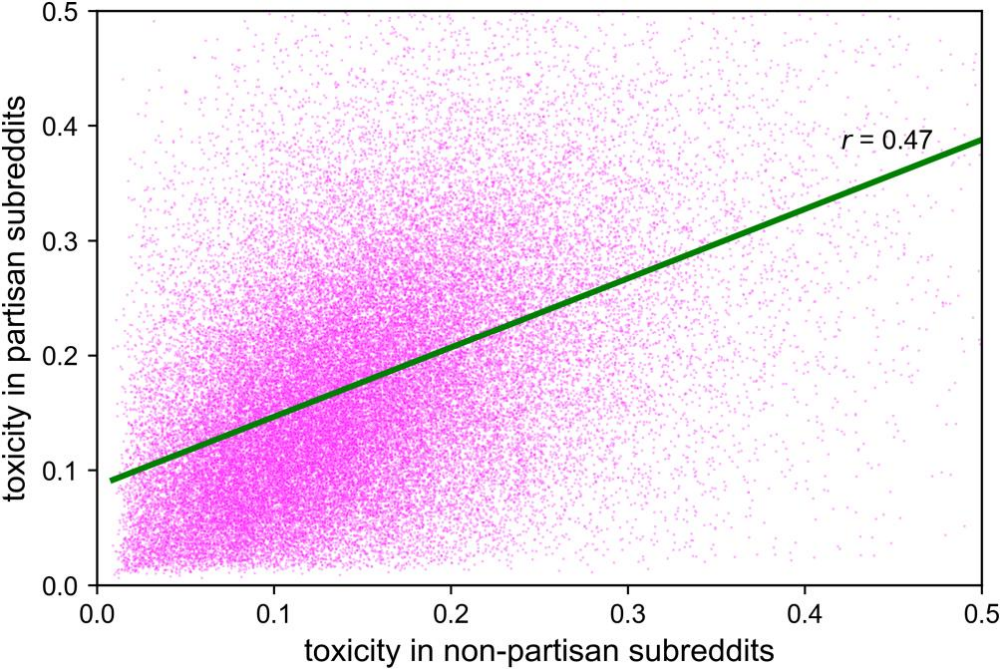
Within-subject correlation of the toxicity of the users with partisan engagement across partisan and nonpartisan subreddits. This random sample of 50,000 engaged users exhibited the same correlation as the full sample of 1,045,631 engaged users (*r* = 0.47).

Such findings are consistent both with prevailing theories (that partisan incivility on social media results from division across ideology or social identity) and with our troll hypothesis (that people who generally behave toxically are especially likely to opt into partisan contexts). But only the troll hypothesis predicts that engaged partisans are toxicity-generalists whose behavior is uncivil even in contexts that are nonpartisan and nonpolitical. As a first test of this idea, we classified as nonpartisan those subreddits with partisan segregation scores within 0.25 *SD*s from the neutral point of 0 (*N_NonpartisanSubreddits_* = 2,084), and as partisan those subreddits with partisan segregation scores at least 2 *SD*s away from that neutral point (*N_PartisanSubreddits_* = 467).^2^ We analyzed toxicity for users who made at least five comments both in partisan and in nonpartisan subreddits within a year of their registration on Reddit (*N_Engaged_* = 1,045,631), excluding comments in nonpartisan subreddits that were classified as political comments (based on the dictionary of Ref. (30)). In support of the troll hypothesis, Fig. 2 reveals that the toxicity these users exhibited in partisan subreddits was highly correlated with their toxicity in nonpartisan subreddits (*r* = 0.47). An auxiliary analysis studying only those users who commented at least 20 times each in partisan and nonpartisan subreddits (i.e. those users for whom we have an especially reliable measure of toxicity) suggests that the actual correlation may be even higher (*r* = 0.60). In short, people are toxic in partisan contexts in large part because they are toxic in general.

As a second test, we focused exclusively on nonpartisan contexts, comparing the commenting behavior of these engaged partisans with that of the nonengaged—users who made at least five comments in nonpartisan subreddits but none in partisan subreddits (*N_NonEngaged_* = 5,255,708). For this comparison, we divided the engaged users into two subgroups: (i) the *unilaterally engaged*, who commented in only left-wing or only right-wing partisan subreddits (*N_Unilaterals_* = 681,311; 57% were left-wing only); and (ii) the *bilaterally engaged*, who commented in both left-wing and right-wing subreddits (*N_Bilaterals_* = 364,320).

**Fig. 3.**
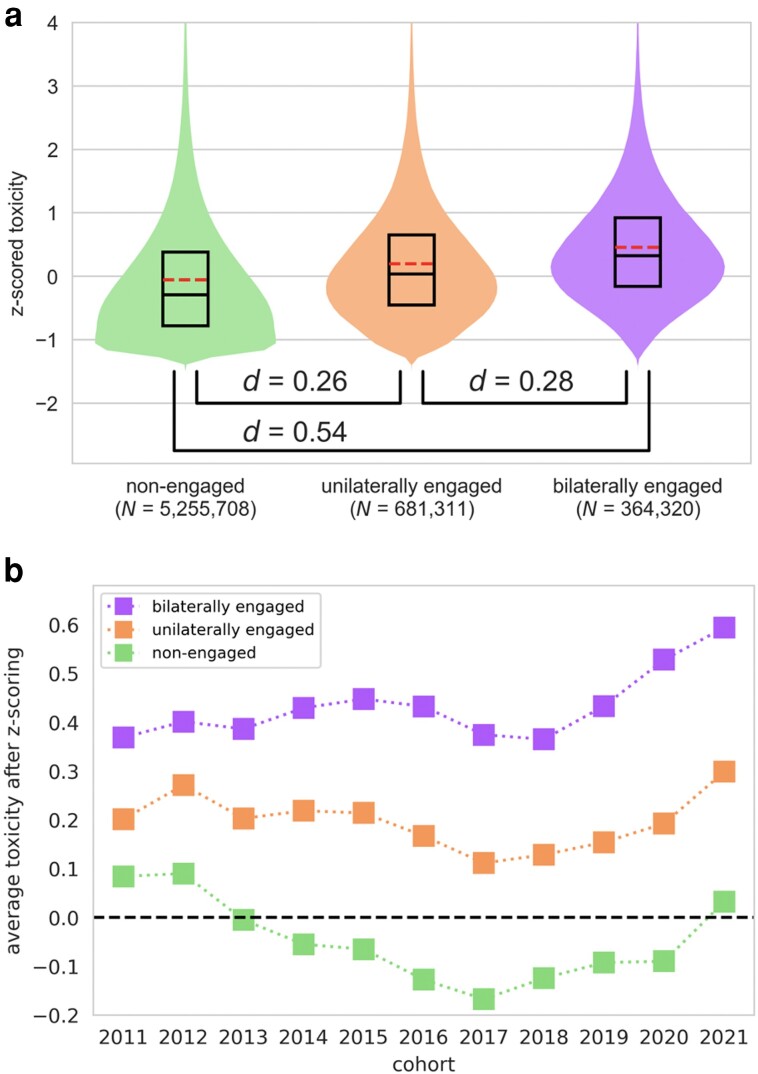
Comparison of the toxicity of the nonengaged, of the unilaterally engaged, and of the bilaterally engaged in nonpartisan subreddits. (a) Violin and box plots. The dashed red lines indicate the means. (b) A cohort corresponds to the year of registration on Reddit.

Figure 3 depicts the toxicity of these three groups in nonpartisan subreddits. Relative to the commenting behavior of the nonengaged (Fig. 3a, green violin plot on the left), the commenting behavior of the unilaterally engaged (Fig. 3a, orange violin plot in the middle) was substantially more toxic (*d* = 0.26). Robustness checks revealed that this effect also emerged for auxiliary measures of incivility (Fig. S1 in *SI Appendix*): Relative to the nonengaged, the unilaterally engaged expressed greater moral outrage (*d* = 0.21) and were less polite (*d* = −0.16) and less prosocial (*d* = −0.17). They were also more profane (*d* = 0.08) and more angry (*d* = 0.09), although those effects were small. In short, when discussing nonpolitical topics in nonpartisan subreddits, the commenting behavior of unilaterally engaged partisans is more uncivil than that of the nonengaged.

What about the bilaterally engaged? Here we consider competing hypotheses. Insofar as toxicity is caused in part by echo-chamber dynamics that prevent social-media users from engaging with opposing partisans, the unilaterally engaged might be more toxic than the bilaterally engaged (*the echo chambers hypothesis*). Alternatively, insofar as people who are generally inclined to engage in toxic discourse seek out highly partisan contexts across the political spectrum, the bilaterally engaged might be even more toxic than the unilaterally engaged (*the bilateral troll hypothesis*).

The results presented in Fig. 3a disconfirm the echo chambers hypothesis and support the bilateral troll hypothesis. Bilaterally engaged partisans (Fig. 3a, purple violin plot on the right) were more toxic than the unilaterally engaged (*d* = 0.28) and far more toxic than the nonengaged (*d* = 0.54). Robustness checks revealed that this tendency for bilaterally engaged partisans to be more toxic than the nonengaged also emerged for the auxiliary measures of incivility (Fig. S1 in *SI Appendix*): Relative to the nonengaged, the bilaterally engaged expressed greater moral outrage (*d* = 0.36), were less polite (*d* = −0.29), and were less prosocial (*d* = −0.31). They were also more profane (*d* = 0.20) and more angry (*d* = 0.15).

The results in Fig. 3a, which emerge across all cohorts of Reddit registrants (see Fig. 3b; the comparison of any two groups within a cohort corresponds to a *P*-value smaller than 10^−47^), provide support for the troll and bilateral troll hypotheses: that engaged partisans (especially the bilaterally engaged) are more uncivil than the nonengaged, even when politics are irrelevant. We subjected these findings to five robustness checks. First, perhaps the results are not about incivility in particular, but about *negativity in general*, including the “internalizing” tendencies of anxiety and sadness (31–33). However, we find that the levels of anxiety and sadness expressed in the comments were nearly identical across the nonengaged, the unilaterally engaged, and the bilaterally engaged (all *d*s < 0.04).

Second, perhaps the toxic behavior of engaged partisans in nonpartisan subreddits results not from a dispositional tendency toward incivility but rather from a socialization process in which engagement in partisan subreddits teaches them uncivil norms, which they then overgeneralize to nonpartisan subreddits (*the socialization hypothesis*). To explore this possibility, we conducted a longitudinal analysis of the users who had partisan engagement. We modeled the toxicity of the comments these users made in nonpartisan subreddits as a function of the partisan activity those users had by the time of posting. A fixed-effects (within) estimator revealed that partisan activity effectively explains 0% of the variance (*R*^2^ < 0.001) of toxicity in nonpartisan subreddits.

**Fig. 4.**
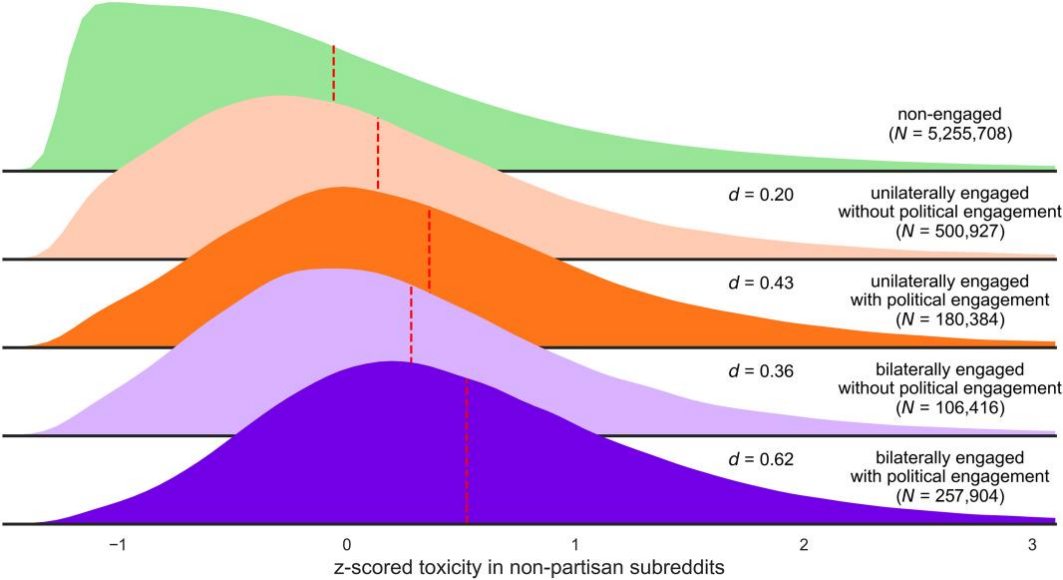
Density plots about the toxicity of five groups of users in nonpartisan subreddits. The dashed red lines indicate the means. Cohen's *d*s are in comparison to the nonengaged.

Third, perhaps the results in Fig. 3 are driven only by users whose engagement in highly segregated subreddits is limited to subreddits of *political content* (e.g. *r/hillaryclinton*, *r/The_Donald*)—or, alternatively, to subreddits that are ostensibly nonpolitical (e.g. *r/librarians*, *r/wrestling*). To consider this possibility, we split the engaged into two groups: those with vs. without any comments in partisan subreddits of political content. As illustrated in Fig. 4, the tendency of the unilaterally engaged and (especially) the bilaterally engaged to be more toxic in nonpartisan subreddits emerged regardless of whether partisans also engaged in partisan subreddits that were explicitly political or ostensibly nonpolitical, but the effects were especially strong for partisans who also engaged in partisan subreddits that were explicitly political. The effect sizes for explicitly political vs. ostensibly nonpolitical subreddits were *d* = 0.43 vs. *d* = 0.20 for the unilaterals and *d* = 0.62 vs. *d* = 0.36 for the bilaterals.

Fourth, we examined whether the observed differences in toxicity are moderated by the *political lean* of the engaged. The users whose partisan engagement was only with left-wing subreddits were virtually exactly as toxic as those whose partisan engagement was only with right-wing subreddits (*d* = 0.27 and *d* = 0.25, respectively, compared to the nonengaged). The bilaterally engaged who commented predominantly in left-wing subreddits (47% of the bilaterally engaged) also exhibited virtually the same level of toxicity as their right-wing counterparts (*d* = 0.56 and *d* = 0.53, respectively, compared to the nonengaged).

Fifth, perhaps the Fig. 3 results were driven by behavior in a small number of *outlier subreddits*, albeit perhaps highly populated ones. To explore this possibility, we considered the 1,221 nonpartisan subreddits in which at least 1,000 comments were posted by each of the three groups (nonpartisans, unilaterals, and bilaterals). The unilaterally engaged were more toxic than the nonengaged in 97% of those subreddits, and the bilaterally engaged were more toxic in 99% of them. We created a subreddit-specific toxicity ratio of the comments made by engaged partisans to the comments made by the nonengaged. Figure S4 in the *SI Appendix* presents a histogram of the results for unilaterals and bilaterals, demonstrating that the average subreddit exhibited a 13.2% toxicity increase for unilaterally engaged partisans relative to the nonengaged (95% confidence interval: 12.7–13.8%) and a 25.6% toxicity increase for bilaterally engaged partisans (95% confidence interval: 24.8–26.5%).

## Discussion

Taken together, the results provide strong and consistent support for the troll hypothesis: (i) people who are especially toxic in partisan contexts are also especially toxic in nonpartisan contexts (Fig. 2), and (ii) engaged partisans (especially the bilaterally engaged) are more toxic than the nonengaged when discussing nonpolitical content in nonpartisan contexts (Figs. 3 and 4). Such effects are specific to uncivil behaviors (rather than to negativity in general) and do not result from some sort of socialization process in partisan subreddits. They emerge regardless of political lean, and they apply to users whose partisan comments take place in contexts that are explicitly political or ostensibly nonpolitical—although they are especially strong for users with activity in explicitly political contexts. The effects, which emerge in virtually all nonpartisan subreddits, help to explain why political contexts tend to be more toxic than nonpolitical contexts (Fig. 1). We conclude that just as people tend to be consistent in their online and offline political behavior (21), they are also consistent in their political and nonpolitical behavior.

Future research will be required to test how strongly these results generalize beyond Reddit. That said, a strength of the present study is that it investigates hundreds of millions of unique behaviors from millions of people across thousands of cultural contexts (subreddits). As such, the results are not subject to the typical concerns about a limited range of cultures or topics of discourse. In addition, social-media environments (e.g. Twitter, Facebook, Reddit) have become a core nexus for political discourse, increasingly functioning as democracy's public square (34). Reddit is a major context where political ideas get introduced and debated—where people of diverse backgrounds and ideologies discuss and argue about which ideas and policies are best (35).

The present findings have important implications for theories of political polarization. They suggest that discourse in partisan contexts is uncivil in large part because the people who opt into it are uncivil. This incivility distorts the public square. People's reluctance to contribute to political discourse—to contribute their views to the marketplace of ideas—is driven less by substantive disagreement than by the tenor of the discourse; they opt out when discourse gets heated (36, 37). It is no wonder that people who are lower in trait hostility tend to opt out of online political discourse (21). The overrepresentation of dispositionally uncivil people in our political discourse is especially troubling because it promotes combative partisanship at the expense of deliberation (38) and leads observers (those who also participate and those who do not) to conclude that the state of our politics is far more toxic than it really is (39).

There is little reason to believe that dispositionally uncivil people have better political ideas than those who are more dispositionally civil, and there is good reason to believe that the uncivil are less prone to compromise, to seek win–win solutions, or to assume that their interlocutors are people of goodwill (8). Consequently, the disproportionate representation of uncivil people in partisan contexts may be a significant contributor to the democratic backsliding afflicting the United States and many other nations in recent years (40). Theories of polarization must engage seriously with the fact that society has built a new megaphone that amplifies the voices of people whose discourse tendencies are disproportionally characterized by toxicity, moral outrage, profanity, anger, impoliteness, and low prosociality.

Past research has demonstrated that passive exposure to social-media posts from opposing partisans can exacerbate polarization (26), but the present study is the first to test whether people who opt into partisan discourse on one vs. both sides of the political divide tend to be especially toxic. Reddit offers its users the opportunity to join multiple communities across the political spectrum, and it gives space for constructive conversations on controversial topics. Nevertheless, our results suggest that this opportunity is exploited by people with especially uncivil tendencies. These findings contribute to an emerging sense of skepticism about whether breaking down echo chambers will reduce polarization or toxicity—at least in a straightforward way. The use of observational data allowed us to identify selection effects related to the behavior of the engaged, but further research is required to establish causal effects.

In contrast to prior research showing that socialization does occur on social media (e.g. Ref. (41)), our results suggest that the incivility of the engaged is not a product of their partisan socialization. However, there are two main aspects in which our work differs from that literature. First, we consider the evolution of toxicity in nonpartisan contexts as a function of the activity in partisan contexts, and not how behavior in partisan contexts is shaped by reinforcement in those same contexts. Second, we consider data from Reddit, where users go by aliases rather than their real names (as on Twitter or Facebook, for example). This heightened anonymity may reduce socialization propensities, an intriguing direction for future research.

Democracy requires conflict. People with differing ideological and policy preferences must compete in the marketplace of political ideas, seeking to persuade others that their own ideas are best. The present research suggests, however, that the voices that are most amplified on social media are dispositionally toxic, an arrangement that seems unlikely to cultivate the sort of constructive discussion and debate that democracies require. The incivility that the engaged partisans exhibit in contexts that are irrelevant to politics raises the concern that toxic behavior in partisan contexts might masquerade as righteousness or advocacy, but it is actually due in large part to these specific people's tendency to be uncivil in general. Consequently, an urgent priority for societies riven by polarization and democratic backsliding is to develop a means of making the public square a congenial environment not only for the dispositionally uncivil but also for people who would be willing to enter the debate if only the tenor of the discourse were less toxic.

## Materials and methods

We used the Pushshift Reddit dataset (42), which includes information about the comments made on Reddit: the author, the posting date, the subreddit, the content, and the unique identifier of a comment. We excluded comments made by users whose username includes the word “bot” and by moderators. PerspectiveAPI has by default a quota limit of 1 query per second. To analyze millions of comments, we made a request for a limit of 1,000 queries per second. This request was approved for a prespecified, limited period.

Our measure of partisan segregation of the subreddits was the absolute value of partisanship derived for 10,006 subreddits by Waller and Anderson (27), who examined all comments on Reddit from 2005 to 2018 to derive a network-based characterization of subreddit partisanship, independent of the content of these comments. This measure of partisanship was *z*-scored, so that the neutral point of 0 corresponded to the average partisanship across the subreddits, and the score of each subreddit was in standard deviation (*SD*) units. The more negative the partisanship of a subreddit, the more left-wing the subreddit, and equivalently for positive-valued (right-wing) subreddits. Because we have defined partisan segregation as the absolute value of this measure, the value 0 remains the neutral point. We categorized subreddits as focusing on either *political* or *nonpolitical* content based on the hierarchical clustering for content-based categorization performed in a separate analysis by Waller and Anderson (27).

In our subreddit-level analysis of the relation between toxicity and partisan segregation (results in Fig. 1), we considered the 9,364 (of the 10,006) subreddits in which at least 10,000 comments were posted from 2011 to 2022 (inclusive). Our available computing resources allowed us to randomly sample for these subreddits a total of 260,425,138 comments (*M* = 27,811, *SD* = 5,725) from that period. We characterized the toxicity of a subreddit by averaging the toxicity of the comments posted in it. Six subreddits with outlier values were excluded from Fig. 1 in the main text to enhance graphical clarity, but no outliers were excluded from the quadratic regression itself.

In our user-level analyses, we considered users who registered on Reddit in the period between 2011 and 2021 (inclusive), and we examined their commenting behavior within a year of their registration (e.g. the commenting behavior of a user who registered on December 31, 2021, would be included through December 31, 2022). The nonengaged are defined as those users with 0 comments in partisan subreddits, and the engaged as those users with at least five comments in partisan subreddits; users with one, two, three, or four comments in partisan subreddits were excluded from our analyses. The consideration of a year from registration allowed us to collect highly rich data, and to assess whether toxicity is dispositional vs. a product of socialization with partisan subreddits (see Figs. S5 and S6 in *SI Appendix* for results based on shorter windows). The number of users for each cohort is presented in Table S1 (*SI Appendix*). We discarded cohorts before 2011 because they lacked enough users (fewer than 100,000 users from 2005 up to 2010, combined) who satisfied our inclusion criterion of having at least five comments in nonpartisan subreddits. In addition, to address the possibility that some political comments might make their way into subreddits that are both nonpolitical in content and nonpartisan in segregation (within 0.25 *SD*s of 0), we discarded all comments in nonpartisan subreddits that include words classified as *issue-based political* by the dictionary-based approach of Simchon, Brady, and Van Bavel (30). For instance, the words “political,” “bipartisan,” “democrat,” “republican,” and “amendment” are some of the words included in this publicly available dictionary. In short, the nonpartisan contexts we study are nonpartisan in triplicate. First, they are nonpartisan according to Waller and Anderson's sociometric measure (within 0.25 *SD*s of the 0 point). Second, they are nonpolitical in content according to Waller and Anderson's content-based clustering. Third, they are nonpolitical at the comment level because they exclude all comments with words of political content as identified by Simchon, Brady, and Van Bavel.

Due to the limitations of our computing resources, for users with more than 120 comments in nonpartisan subreddits (8% of the users), we randomly sampled 120 of their comments. Similarly, for the users with partisan engagement who have more than 120 comments in partisan subreddits (9% of engaged), we randomly sampled 120 of their comments in these contexts. The toxicity of a user was derived by averaging the toxicity of the user's comments. Of the 1,045,631 engaged users, 310,830 (30%) made at least 20 comments in both partisan and nonpartisan subreddits (these users are included in the reported auxiliary analysis about the within-subject correlation of the engaged).

In the model developed for the second robustness check (testing the socialization hypothesis), we included three predictors tapping partisan activity by the time of comment-posting in a nonpartisan subreddit. The first two were dichotomous: whether the user already had (i) unilateral and (ii) bilateral partisan engagement. The third was continuous: (iii) the number of comments in partisan subreddits the user had made. Because the number of comments in partisan subreddits exhibited a right-skewed distribution across the engaged (see Table S6 and Fig. S9 in *SI Appendix*), we added to the model the following three transformations of the continuous predictor: (iv) its logarithm, (v) its square root, and (vi) its cubic root. Thus, the model developed for the second robustness check had six predictors in total. In the fifth robustness check, the 95% confidence intervals about the subreddit toxicity increase for the users with partisan engagement were bootstrapped (10,000 repetitions).

In addition to the toxicity of PerspectiveAPI, we also assessed several additional measures that are arguably proxies for incivility. The *moral outrage* of the comments was assessed with the classifier of Brady et al. (41). This classifier assesses the probability that a comment expresses feelings in response to a violation of moral norms, and where these feelings are comprised of emotions such as anger, disgust, and contempt. For *profanity*, *anger*, *politeness*, *prosociality*, *anxiety*, and *sadness*, we employed the dictionary-based approach of the Linguistic Inquiry and Word Count (LIWC (43)). Because this approach can be executed only in a centralized fashion, which makes difficult the assessment for comments whose number is in the hundreds of millions, we developed our own dictionary-based method by reverse-engineering LIWC. We purchased a LIWC license and analyzed over 760,000 unique words with that official software. The results in Table S2 (*SI Appendix*) demonstrate that our dictionary method provides a very close approximation of LIWC. We evaluated the comments of the users with our dictionaries and then characterized the users based on the averages of their comments.

## Supplementary Material

pgad325_Supplementary_Data

## Data Availability

The data are available at https://osf.io/6bs2v/

## Footnotes

1
https://www.semrush.com/blog/most-visited-websites/

2 To meet our inclusion criteria for establishing a context as nonpartisan, we excluded 16 nonpartisan subreddits that were explicitly political (0.76% of the nonpartisan subreddits). For the partisan subreddits, 105 (22.48%) were explicitly political; later, we report results separately for partisan subreddits of political vs. nonpolitical content.
