# Supplementary material for: The social media discourse of engaged partisans is toxic even when politics are irrelevant
Source: PNAS Nexus. 2023 Oct 6;2(10):pgad325. doi: 10.1093/pnasnexus/pgad325 (PMC10588776; doi:10.1093/pnasnexus/pgad325)

**The Social Media Discourse of Engaged Partisans is Toxic Even when Politics are Irrelevant**

**Supplementary Information**

Table of Contents

[A. Number of users 2](#_Toc145408032)

[B. Auxiliary measures of incivility 3](#_Toc145408033)

[C. Number of comments in non-partisan subreddits 5](#_Toc145408034)

[D. Users with exactly one comment in partisan subreddits 7](#_Toc145408035)

[E. Figure corresponding to the fifth robustness check in the main text 8](#_Toc145408036)

[F. First comment in non-partisan subreddits 9](#_Toc145408037)

[G. Alternative time-windows 11](#_Toc145408038)

[H. Further results for the first subreddit-level analysis 14](#_Toc145408039)

[I. Second robustness check 16](#_Toc145408040)

# Number of users

In Table S1 we present for each cohort the number of users in our sample. The results in Figures 2, 3, and 4 of the main text were derived by analyzing the commenting behavior these users exhibited within a year from their registration on Reddit.

Table S1. The number of users per cohort.

| cohort | non-engaged | unilaterally engaged | bilaterally engaged |
| --- | --- | --- | --- |
| 2011 | 149,559 | 16,949 | 7,915 |
| 2012 | 268,120 | 34,324 | 13,336 |
| 2013 | 275,149 | 30,391 | 12,338 |
| 2014 | 324,920 | 34,055 | 14,047 |
| 2015 | 367,536 | 35,819 | 21,392 |
| 2016 | 461,451 | 49,190 | 38,329 |
| 2017 | 353,968 | 48,259 | 38,212 |
| 2018 | 413,110 | 76,287 | 47,244 |
| 2019 | 665,608 | 107,643 | 55,329 |
| 2020 | 1,033,400 | 134,786 | 66,723 |
| 2021 | 942,887 | 113,608 | 49,455 |
|  |  |  |  |
| Total  *N* = 6,301,339 | 5,255,708 | 681,311 | 364,320 |

# Auxiliary measures of incivility

The results in Table S2 demonstrate that our dictionary method is a very close approximation of LIWC.

Table S2. The correlation between LIWC and our dictionaries evaluated on a random sample of 20,000 Reddit comments.

| profanity | .99 |
| --- | --- |
| anger | .94 |
| politeness | .95 |
| prosociality | .97 |
| anxiety | .99 |
| sadness | .95 |

In Figure S1 we present a graphical representation of the results reported in the main text about the auxiliary measures of incivility. In Figure S2, we present the correlations among the different behavioral measures based on the commenting behavior of the users in non-partisan subreddits. Our incivility measure was highly correlated with moral outrage and profanity (*r* = ~|.5|), moderately correlated with politeness and prosociality (*r* = ~|.2|), and slightly correlated with anger (*r* = ~|.1|). As anticipated, it was largely uncorrelated with anxiety and sadness (*r* = ~|.0|).

Figure S1. Comparison of different measures of incivility of the non-engaged, of the unilaterally engaged, and of the bilaterally engaged in non-partisan subreddits. Positive values of Cohen’s *d* correspond to greater values for the users with partisan engagement.


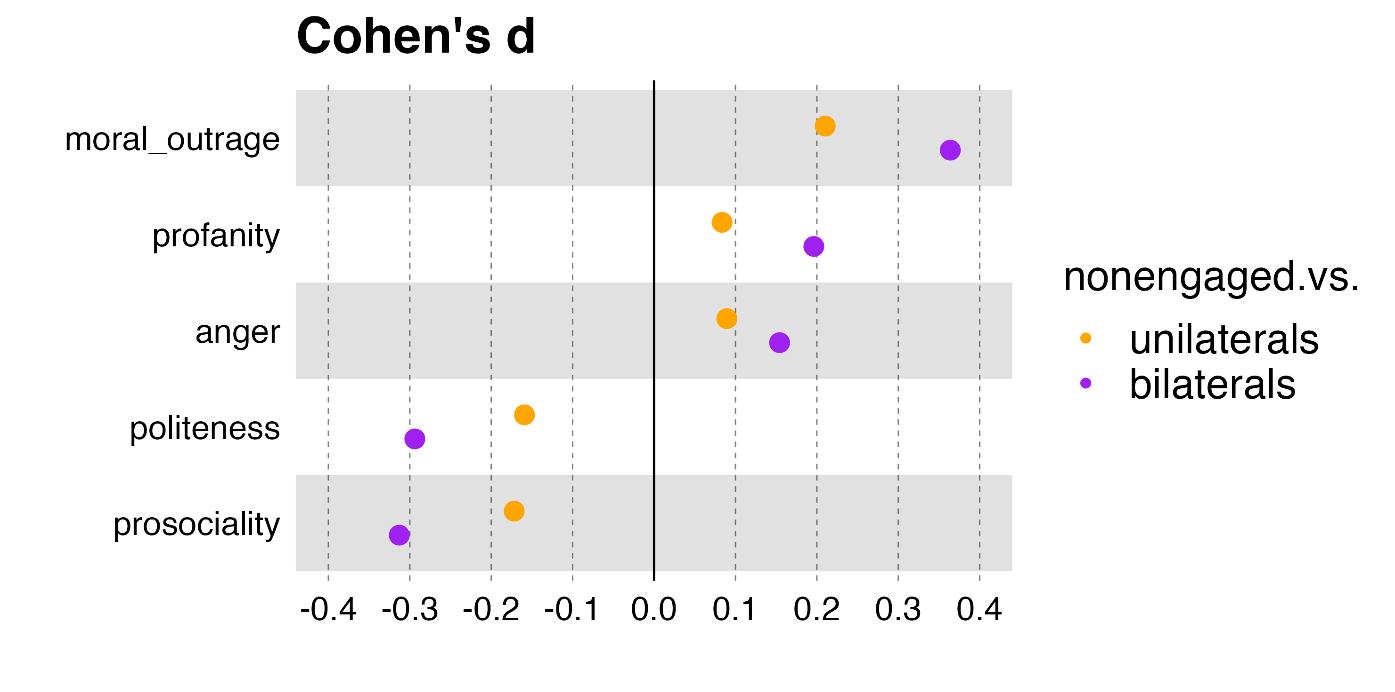


Figure S2. The correlation of behavioral measures as assessed for users based on their comments in non-partisan subreddits.


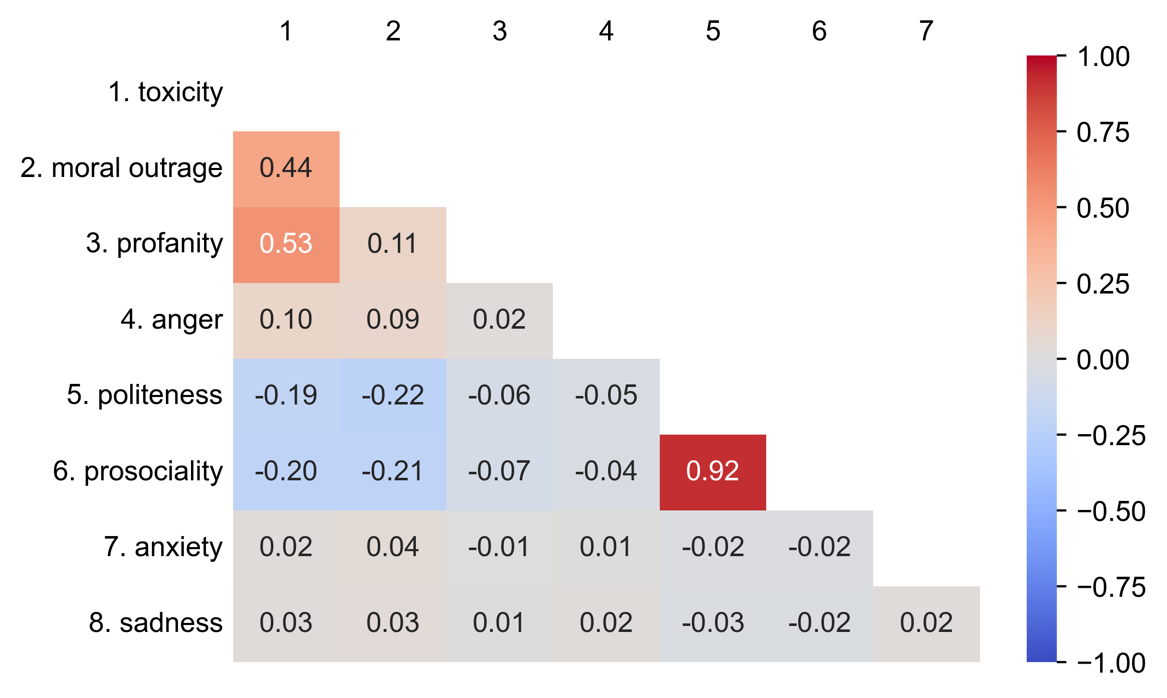


# Number of comments in non-partisan subreddits

The analysis in the second robustness check (testing the socialization hypothesis) involved the number of comments that engaged partisans made in partisan subreddits. Here, we first report summary statistics for the number of comments that each group made in non-partisan (Table S3).

In Table S3 we observe that in non-partisan subreddits, the bilaterally engaged users made on average more comments than the unilaterally engaged users (*M* = 130 vs. 74), who in turn made more comments than the non-engaged (*M* = 34). Therefore, it is possible that the heightened toxicity (average toxicity over comments) of the engaged is explained by their high levels of activity in non-partisan subreddits, rather than by the fact that they have opted into partisan contexts. The results in Table S4 reject this possibility. In Column A, the toxicity in non-partisan subreddits was regressed on two binary predictors, one each for unilateral and bilateral partisan engagement. When the number of comments in non-partisan subreddits was also included in the predictors (Column B), the estimates for the coefficients of the two binary predictors changed only marginally, from 0.0226 to 0.0224 for the unilaterals and from 0.0456 to 0.0450 for the bilaterals. A similar observation was made when the number of comments in partisan subreddits was also included in the regression (Column C), as expected based on the results of the second robustness check (which involved only the engaged). These results imply that the increased toxicity the engaged exhibited in non-partisan subreddits is not explained by their number of comments in partisan or non-partisan subreddits. Also, the results in Column A imply that the average toxicity for the non-engaged was 0.121, for the unilaterally engaged 0.143, and for the bilaterally engaged 0.166.

Table S3. Comments in non-partisan subreddits.

|  | Mean | SD | Median |
| --- | --- | --- | --- |
| non-engaged | 34 | 162 | 12 |
| unilaterally engaged | 74 | 210 | 26 |
| bilaterally engaged | 130 | 384 | 53 |

Table S4. Regression results for toxicity in non-partisan subreddits. Controlling for the number of comments does not change the correlation between toxicity and partisan engagement.

|  | (A) | (B) | (C) |
| --- | --- | --- | --- |
|  |  |  |  |
| intercept | 0.1211 | 0.1209 | 0.1209 |
| unilaterally engaged | 0.0226 | 0.0224 | 0.0221 |
| bilaterally engaged | 0.0456 | 0.0450 | 0.0445 |
| number of comments in non-partisan subreddits | - | 0 | 0 |
| number of comments in partisan subreddits | - | - | 0 |

# Users with exactly one comment in partisan subreddits

We also examined whether having just one comment in partisan subreddits was sufficient to predict toxicity in non-partisan subreddits. The results in Figure S3 reveal that this is the case: In predicting behavior in non-partisan subreddits, users with exactly one comment in partisan subreddits were more toxic than those with zero comments in partisan subreddits (*d* = 0.23).

Figure S3. Comparison of toxicity in non-partisan subreddits. The users with exactly one comment are not part of any of our other analyses.


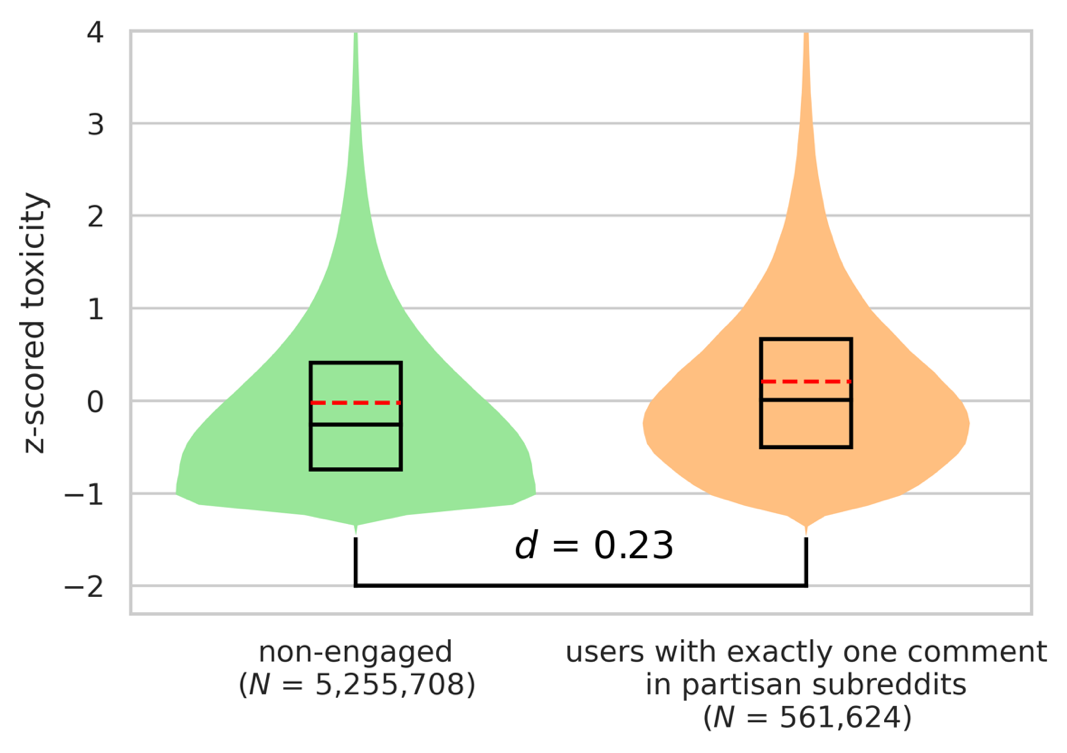


# Figure corresponding to the fifth robustness check in the main text

Figure S4. Frequency of non-partisan subreddits based on the toxicity-ratio of the comments made by engaged partisans to the comments made by the non-engaged.


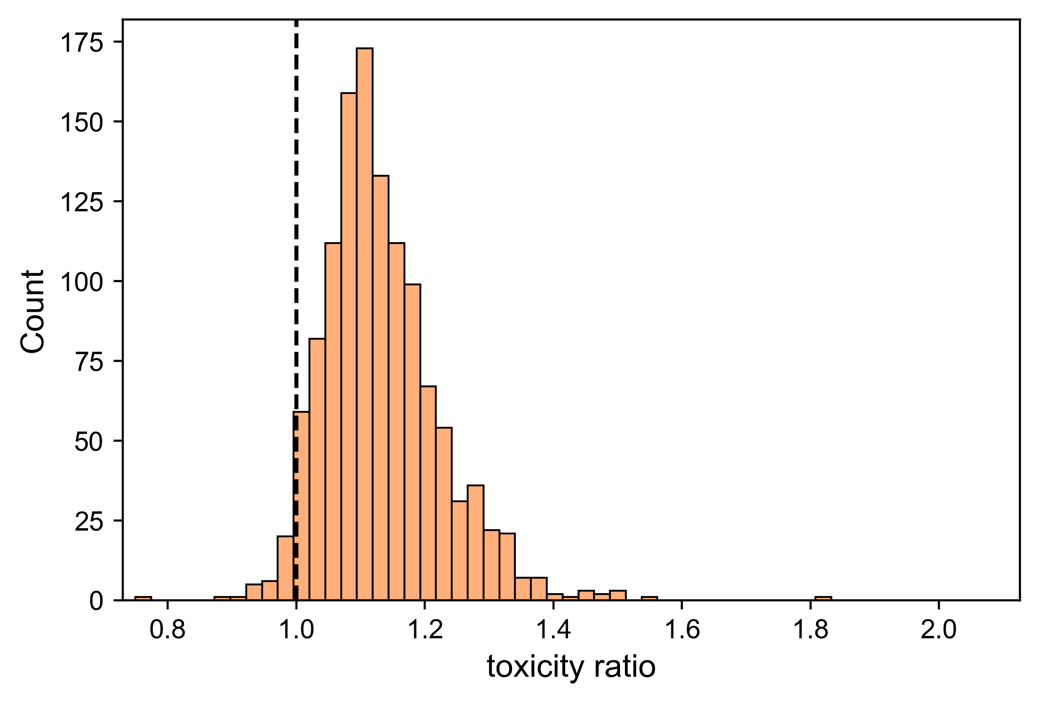


(a) unilaterally engaged vs. non-engaged


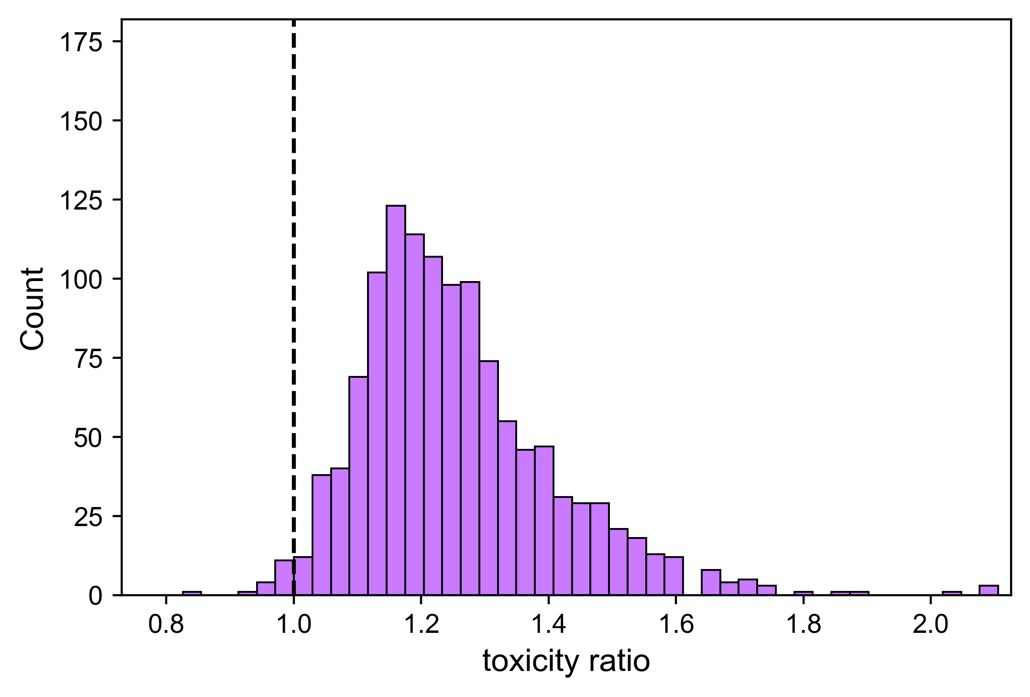


(b) bilaterally engaged vs. non-engaged

# First comment in non-partisan subreddits

This section presents the results of another analysis that provides further support for the troll hypothesis, focusing solely on the commenting behavior of the users with partisan engagement. If the troll hypothesis is correct, the toxicity of the first comment of the engaged partisans in non-partisan subreddits should correlate with the toxicity of their subsequent comments in non-partisan subreddits, and it should not correlate with their partisan activity before the time of that first comment.

Table S5 presents the results of two regressions whose dependent variable is the toxicity of the first comment the (engaged) users made in non-partisan subreddits. The predictors of the regression corresponding to column (A) consist of a dummy variable modeling whether a comment in partisan subreddits had been made before the first comment in non-partisan subreddits, the number of comments in partisan subreddits before the first comment in non-partisan subreddits, and the cohort of the users. We observe that the predictors explain 0% of the variance (*R*^2^ = 0) of the toxicity of the first comment in non-partisan subreddits. Therefore, in line with the troll hypothesis, the toxicity of the first comment in non-partisan subreddits is uncorrelated with the prior activity of the users in partisan subreddits (and with their cohort).

When the average toxicity of a user’s subsequent comments (excluding the first comment) in non-partisan subreddits is included in the predictors, we observe that the proportion of the variance that the model explains increases from 0% (column A) to 6.4% (column B). This result implies that the first comment of the (engaged) users in non-partisan subreddits is indeed correlated with their overall toxicity. Notice that a single comment can provide noisy information, and thus a correlation with overall behavior could have not been detected, even if it existed. The fact that such a correlation is found even when only one comment is considered is indicative of the validity of the troll hypothesis.

Table S5. Regression results for the toxicity of the first comment of the engaged partisans (*N* = 1,045,630) in non-partisan subreddits.

|  | (A) | (B) |
| --- | --- | --- |
|  |  |  |
| intercept | 0.1469 | 0.0556 |
| had a comment in partisan subreddits before the first comment in non-partisan subreddits | 0.0050 | 0.0048 |
| comments in partisan subreddits before the first comment in non-partisan subreddits | 0 | 0 |
| subsequent toxicity in non-partisan subreddits | - | 0.6127 |
|  |  |  |
| *R*^2^ | 0 | 0.064 |

Note. Predictors for the cohort of the users are included in both regressions.

# Alternative time-windows

All of the results presented so far are based on the behavior of users within a year from their registration on Reddit. The choice of this time-window allowed us to collect a highly rich sample, while the possibility that the toxicity of the engaged partisans is a product of socialization with partisan contexts was rejected in our second robustness check. To further assess that our findings are not tied to the chosen time-window, we also considered shorter time-windows (2 and 6 months). which can provide further evidence in favor of a dispositional interpretation of our results, while we kept all the other inclusion criteria the same (as described in the main text).

The consideration of a time-window of 2 months resulted to a sample consisting of 222,868 engaged partisans, and of a time-window of 6 months to a sample of 573,618 engaged partisans. These sample sizes are considerably lower than the size of the sample considered in the main text (*N_Engaged_* = 1,045,631). Yet, as the results in Figure S5 reveal, the within-subject correlations (*r* = .44 for the time-window of 2 months; *r* = .46 for the time-window of 6 months) were effectively the same to the corresponding correlation presented in the Figure 2 of the main text (*r* = .47). Moreover, the results in Figure S6 about the comparisons of the three groups (non-engaged, unilaterals, and bilaterals) were very close to the corresponding results in the main text. More specifically, the difference in the toxicity of the non-engaged and of the unilaterally engaged corresponded to *d* = 0.31 for the 2-months window and to *d* = 0.28 for the 6-months window, and thus these differences are qualitatively the same to the corresponding difference for the 1-year window (*d* = 0.26). The comparison between the non-engaged and the bilaterals led to the same conclusions (*d* = 0.58 for the 2-months window, *d* = 0.56 for the 6-months window, *d* = 0.54 for the 1-year window). Therefore, the consideration of a 1-year window from the time of registration allowed the examination of a large sample without diluting our findings.

Figure S5. Within-subject correlation of the toxicity of the users with partisan engagement across partisan and non-partisan subreddits for different time-windows. Each of these random samples of 50,000 engaged users exhibited the same correlation as their corresponding full samples.


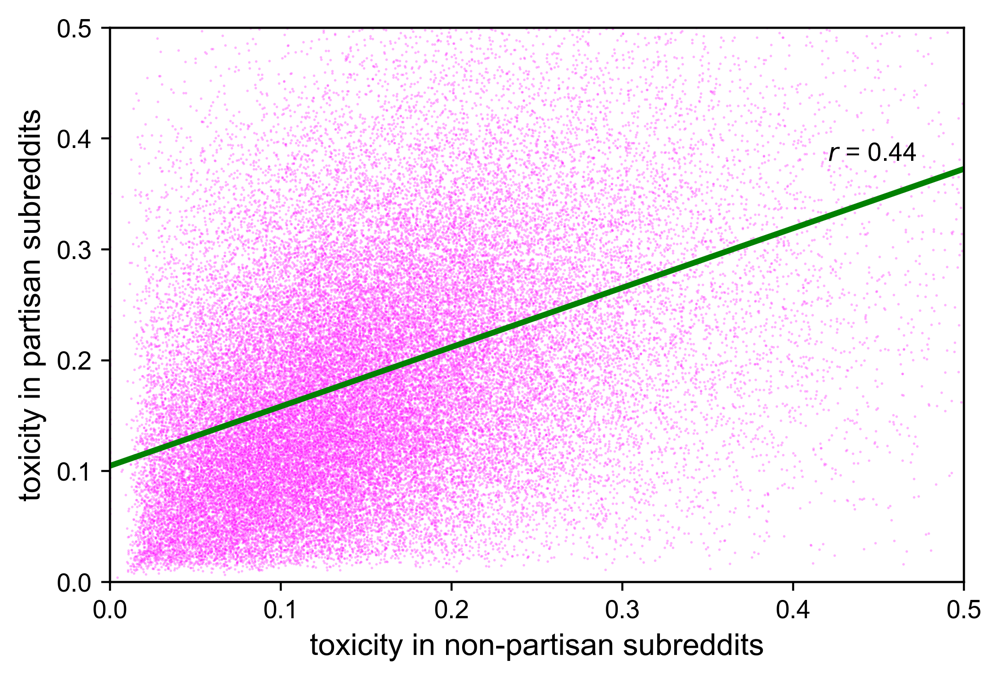


(a) Time-window: 2 months from registration


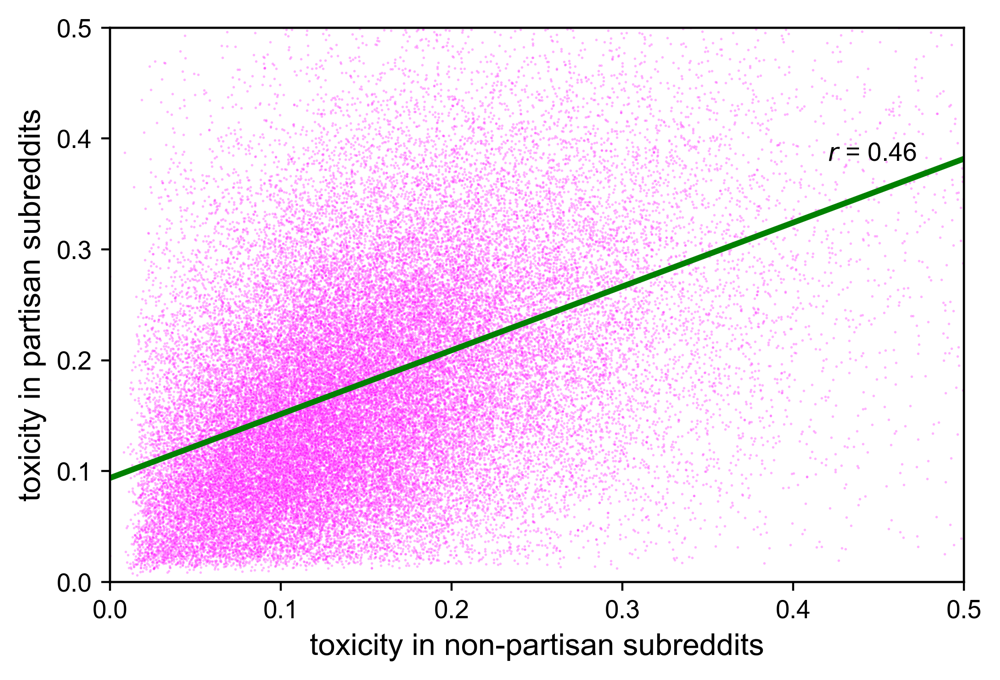


(b) Time-window: 6 months from registration

Figure S6. Violin and box plots for the comparison of the toxicity of the non-engaged, of the unilaterally engaged, and of the bilaterally engaged in non-partisan subreddits, for different time-windows. The dashed red lines indicate the means.


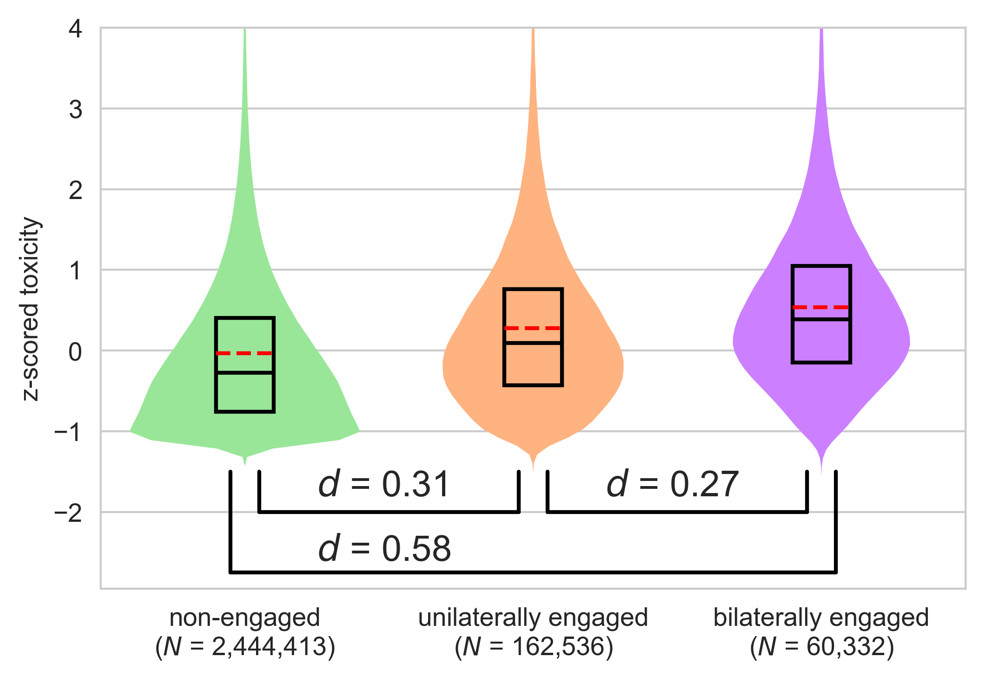


(a) Time-window: 2 months from registration


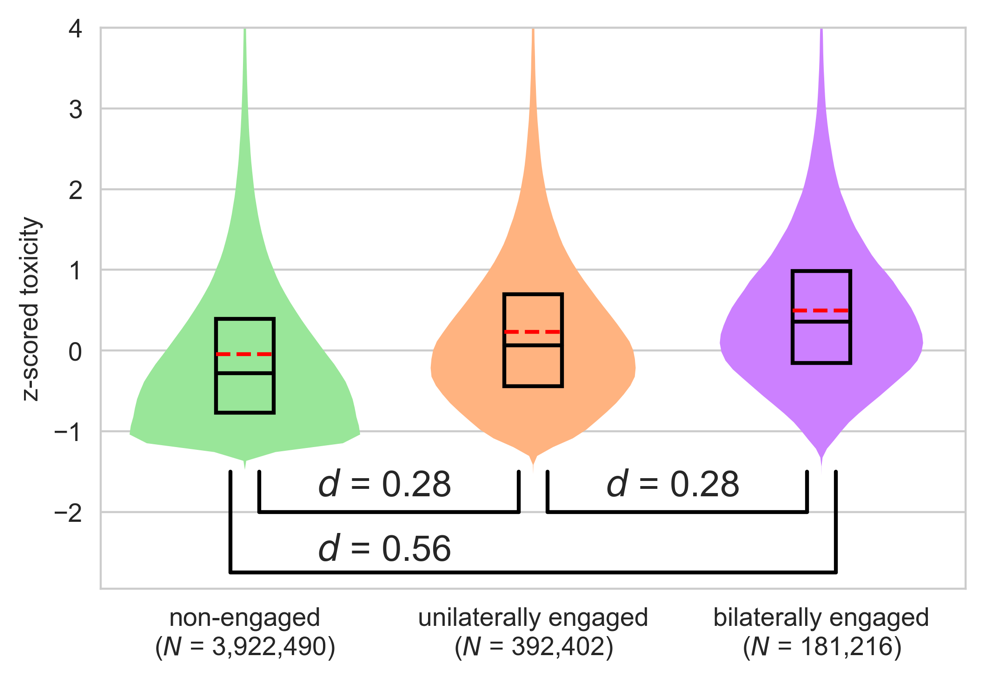


(b) Time-window: 6 months from registration

# Further results for the first subreddit-level analysis

In the main text, we examined the association between the toxicity and the partisan segregation of the subreddits (Figure 1). Here, we report additional results about the association of these two measures. While the quadratic regression of toxicity on partisan segregation revealed a significant quadratic effect (*β* = .21, *p* < .0001), the linear term of this regression was not significant at *p* = 0.05 (*β* = −.04, *p* = .10). The Pearson correlation between toxicity and partisan segregation was *r* = .15 (*p* < .0001), and the Spearman correlation was *ρ* = .10 (*p* < .001).

To further assess the robustness of the findings from the quadratic regression, we also considered a non-parametric regression (locally weighted linear regression) of toxicity on partisan segregation. As observed in Figure S7, the results of the two regressions are in high agreement for the vast majority of the values of partisan segregation. Only for very high values of partisan segregation (> 4), involving only a handful of subreddits, the results of the two regressions diverged.

Figure S7. Quadratic and non-parametric regression of the toxicity of 9,364 subreddits on their partisan segregation.


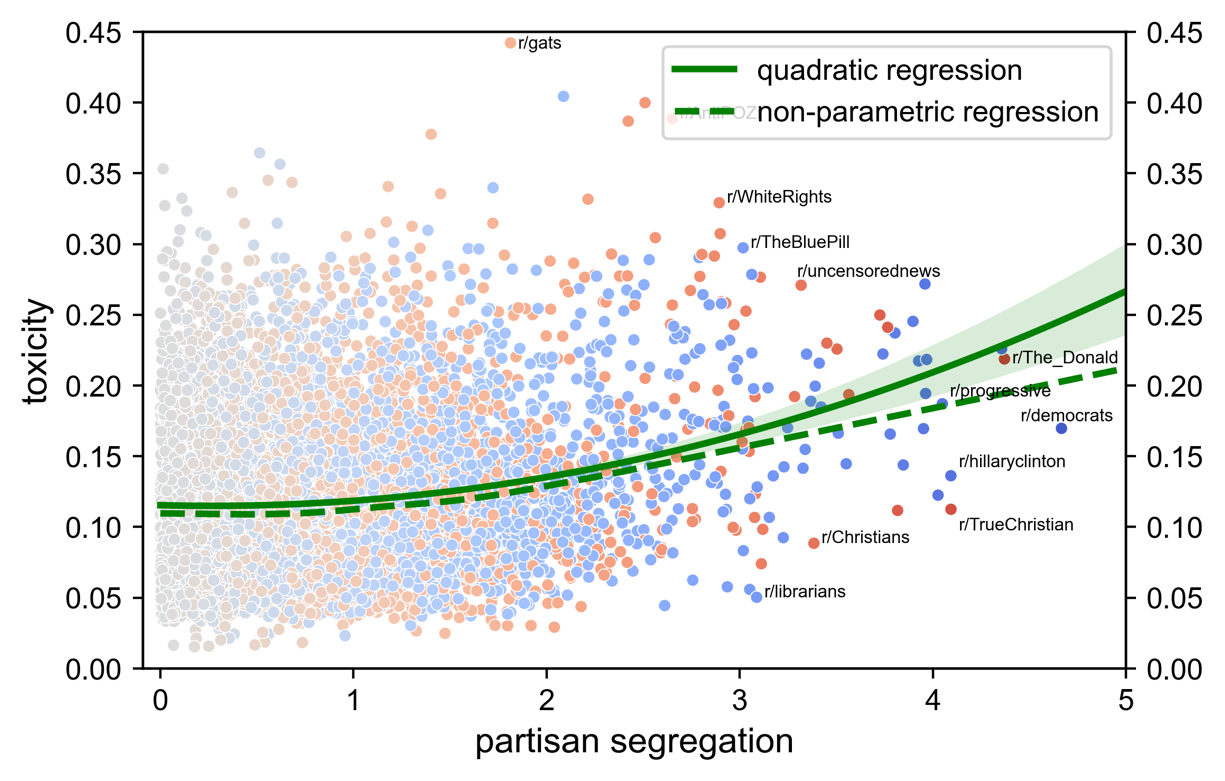


Moreover, to examine whether the results of the quadratic regression were moderated by partisan side, we conducted separate quadratic regressions for left-wing and for right-wing subreddits. For the left-wing subreddits, the quadratic effect was *β* = .20 (p < .0001), and the linear effect *β* = −.01 (*p* > .82). For the right-wing subreddits, the quadratic effect was *β* = .21 (p < .0001), and the linear effect *β* = −.07 (*p* < .04). Therefore, the quadratic effect was virtually the same in the regressions of left-wing and right-wing subreddits. The results of these two quadratic regressions are also depicted in Figure S8.

Figure S8. The toxicity and partisan segregation of left-wing and of right-wing subreddits.


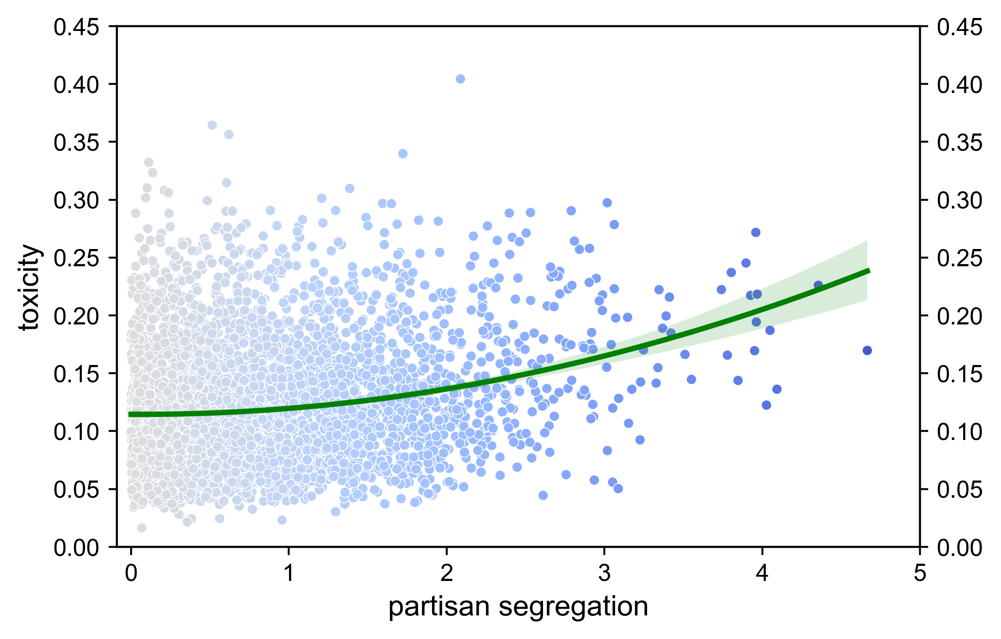


(a) Left-wing subreddits


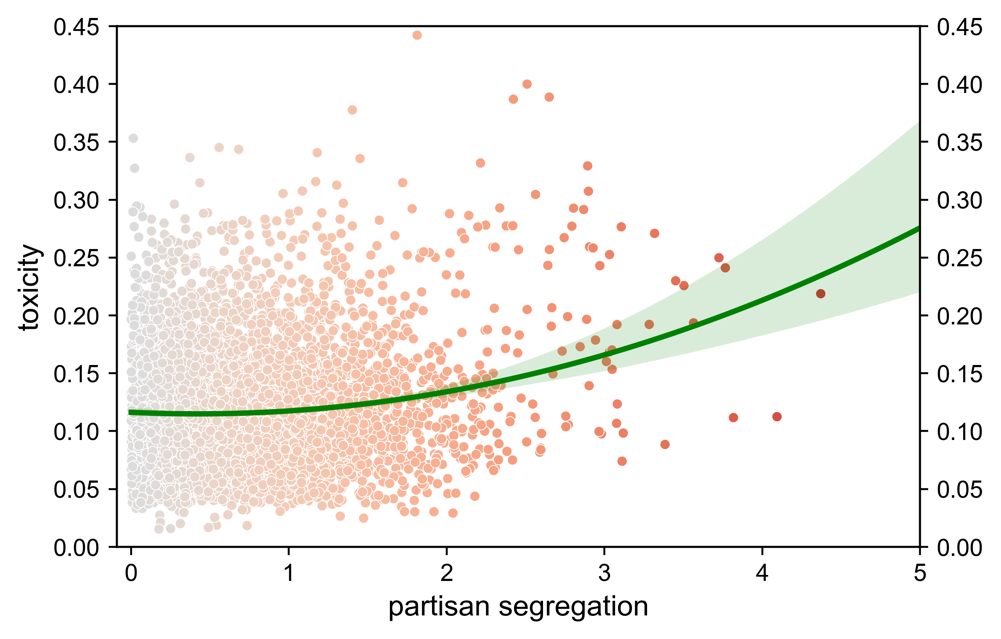


(b) Right-wing subreddits

# Second robustness check

In the second robustness check, we conducted a longitudinal analysis to assess whether the incivility of the engaged partisans is a product of their socialization with partisan subreddits. This analysis modeled the toxicity of each comment that the engaged had in non-partisan subreddits as a function of their partisan activity by the time of comment-posting (in non-partisan subreddits). The fact that we employed a fixed-effects (within) estimator implies that the model also included user-specific intercepts which were not estimated. The specification of the model described in the Materials and Methods section of the main text is,

$$toxicity\text{\_}non\text{\_}partisan\text{\_}comment_{it}=\alpha+\beta has\text{\_}partisan\text{\_}comment_{it}+\gamma has\text{\_}bilateral\text{\_}engagement_{it}+\delta number\text{\_}of\text{\_}partisan\text{\_}comments_{it}+\zeta\log\left( number\text{\_}of\text{\_}partisan\text{\_}comments_{it}+1 \right)+\theta\sqrt{number\text{\_}of\text{\_}partisan\text{\_}comments_{it}}+\kappa\sqrt[3]{number\text{\_}of\text{\_}partisan\text{\_}comments_{it}}+\eta_{i}+\epsilon_{it}$$

where $number\text{\_}of\text{\_}partisan\text{\_}comments_{it}$ is the number of comments in partisan subreddits that user $i$ had by the time of posting their $t^{\text{th}}$ comment in non-partisan subreddits, $\eta_{i}$ is the user-specific intercept (which does not depend on the time of comment-posting), and $\epsilon_{it}$ is an error term.

The three transformations of the $number\text{\_}of\text{\_}partisan\text{\_}comments_{it}$variable (logarithm, square root, cubic root; the constant 1 was added in the logarithmic transformation because a user could have 0 comments in partisan subreddits by the time of posting their $t^{\text{th}}$ comment in non-partisan subreddits) were added to the model because, as presented in column A of Table S6, the distribution of the number of partisan comments over the engaged was highly right-skewed (skewness = 22.99). Even the distribution of the logarithm of this variable was highly right-skewed (skewness = 1.11), albeit much less so. These observations imply that there are some outlier users with an extremely high number of comments in partisan subreddits compared to the rest of the engaged. Therefore, the extremely low *R*^2^ (< .001) derived by the fixed-effects estimator could have been due to a very small subset of the users who contribute many data points but whose (lack of) socialization propensity might be very different to that of the other users.

Columns B, C, and D of Table S6 present results after the exclusion of the top 1%, 25%, and 50% (correspondingly) of the engaged with respect to their number of comments in partisan subreddits. That is, column D, for instance, presents results for the 50% of the users with the lowest number of comments in partisan subreddits. We observe that the distribution of this variable becomes significantly less skewed as the exclusion criterion becomes more stringent. For instance, for the bottom 50% of the engaged (with respect to their comments in partisan subreddits), the skewness of the distribution of the number of comments in partisans subreddits was 0.55, and the skewness of the distribution of the logarithm of this variable was only 0.16. The observation that the skewness issue gets increasingly mitigated by the logarithmic transformation as the exclusion criterion becomes more stringent is also offered by Figure S9, which pictorially presents the distribution of the logarithm of the number of comments in partisan subreddits.

Having established that we can mitigate the skewness of the number of comments in partisan subreddits (across the engaged, by excluding outlier users), which is included in the predictors of the model (in a time-dependent fashion), we will now present results that further assess the finding of the longitudinal analysis with the fixed-effects (within) estimator where all users with partisan engagement were included (*R*^2^ < .001). We repeated this longitudinal analysis three times, excluding the users with the top 1% of comments in partisan subreddits in the first repetition, the users with the top 25% in the second repetition, and the users with the top 50% in the third repetition. In each of these analyses, the predictors about partisan activity again explained essentially 0% of the variance (*R*^2^ < .001) of the toxicity of the comments in non-partisan subreddits. Therefore, even when a predictor about the number of comments in partisan subreddits was not highly right-skewed across the engaged (e.g., skewness = 0.16 for the bottom 50% of the engaged), partisan activity was essentially uncorrelated with toxicity in non-partisan subreddits.

We conclude that the toxicity that the engaged exhibit in non-partisan subreddits is not a product of their partisan activity, as the findings of the second robustness check were replicated even when outlier users were excluded from the analysis.

Table S6. Summary statistics about the number of comments in partisan subreddits based on various exclusion criteria for the users with partisan engagement.

|  | (A)  No  exclusions  (*N*_users_ = 1,045,630) | (B)  Excluding  the top 1%  (*N*_users_ = 1,035,174) | (C)  Excluding  the top 25%  (*N*_users_ = 780,033) | (D)  Excluding  the top 50%  (*N*_users_ = 516,976) |
| --- | --- | --- | --- | --- |
| number of comments in partisan subreddits | | | | |
| Mean | 54.15 | 39.92 | 13.21 | 8.25 |
| SD | 201.51 | 73.78 | 8.14 | 2.77 |
| Skewness | 22.99 | 4.34 | 1.07 | 0.55 |
| logarithm of the number of comments in partisan subreddits | | | | |
| Mean | 2.97 | 2.92 | 2.41 | 2.06 |
| SD | 1.17 | 1.09 | 0.58 | 0.33 |
| Skewness | 1.11 | 0.90 | 0.30 | 0.16 |

Figure S9. Distribution of the users with partisan engagement based on the logarithm of their number of comments in partisan subreddits, excluding the top 0%/1%/25%/50% of these users with respect to their number of comments in partisan subreddits.


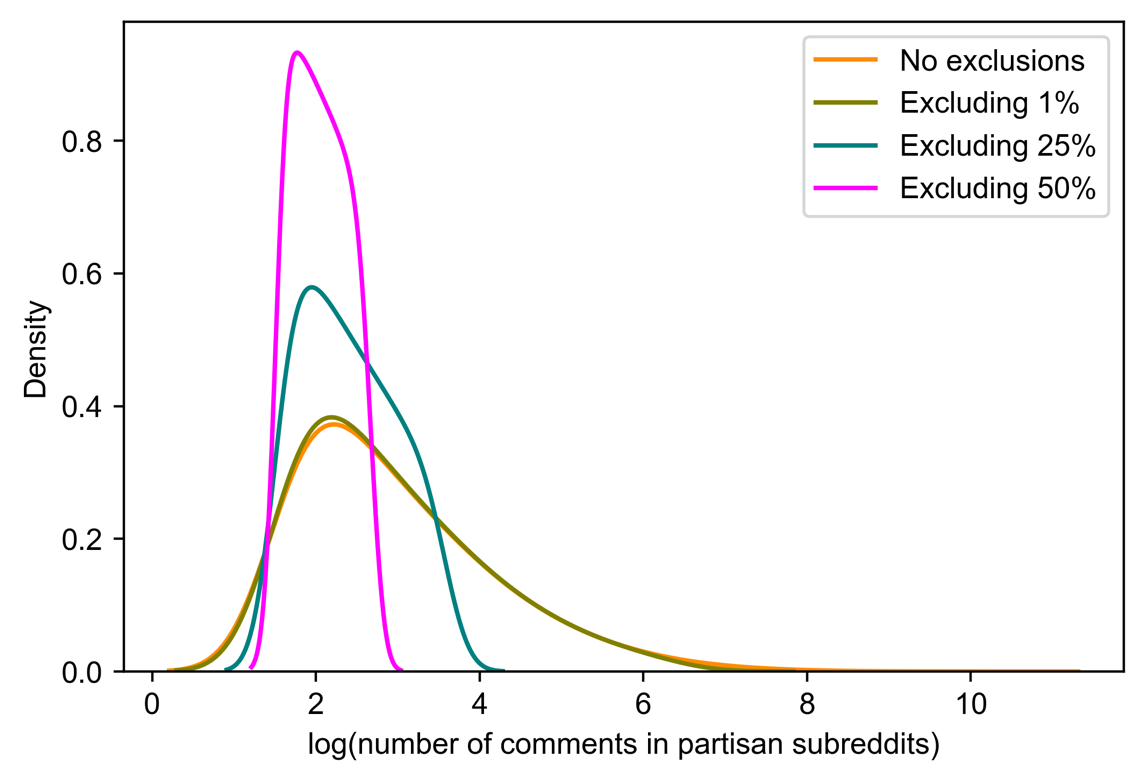

Supplement: pgad325_Supplementary_Data [file pgad325_supplementary_data.docx]
